# Community‐based tourism, peasant agriculture and resilience in the face of COVID‐19 in Peru

**DOI:** 10.1111/joac.12447

**Published:** 2021-08-31

**Authors:** Jordi Gascón, Kevin S. Mamani

**Affiliations:** ^1^ Department of Social Anthropology University of Barcelona Barcelona Spain; ^2^ Qhapaq Ñan Project Ministry of Culture Lima Peru

**Keywords:** Andes, community‐based tourism, peasant agriculture, pluriactivity, resilience, subsistence agriculture

## Abstract

In the Andes, the diversification of economic activities among the peasant population is common practice. However, it is not a uniform strategy: as new employment and economic possibilities have emerged, the disparity of pluriactive strategies has multiplied. Based on a particular case study (Amantaní Island, Lake Titicaca), where community‐based tourism has developed strongly, we will compare the resilience of these strategies. The COVID‐19 pandemic, which paralysed economic activities, highlighted that the least vulnerable pluriactive strategies were those that included subsistence agriculture. In fact, this is something that the peasant population itself perceives: although the role of this type of agriculture in the family economy is decreasing, most households still invest time and capital to increase their family's agricultural resources.

## 1 INTRODUCTION

The definition of a peasant farmer as a solely or eminently agricultural producer was questioned in the 1980s (Marsden, 1990; Shanin, 1986). But it was in the 1990s that researchers understood the need to systematically analyse peasant farmers' participation in nonagrarian activities in order to understand their economic strategies (Ellis, 1998). In Latin American rural studies, the phenomenon of pluriactivity was initially considered a consequence of the interests of an underdeveloped capitalist market. According to this idea, so that domestic production can compete with imports, labour cost must go down. This is possible if the worker covers part of his reproduction costs autonomously through subsistence agriculture. Due to poverty, peasants are forced to participate in a labour market that pays them low wages and does not offer them the option of proletarianisation—that is, to abandon subsistence farming and live solely on wages (e.g., Caballero, 1990; de Janvry et al., 1989; for the Andes, Gonzales de Olarte, 1994; Loker, 1996; Mayer, 1994).

But it was later observed that pluriactivity was not so much the result of market dynamics on submissive peasant farmers as a strategy applied by entrepreneurial peasant farmers taking advantage of all the economic options available to them, which included what the capitalist market could offer (de Grammont & Martínez Valle, 2009; Martínez Valle, 2021). Furthermore, it was seen that pluriactivity was not necessarily a consequence of poverty: wealthier peasant farmers diversified their economy by investing capital and labour in activities outside their farm without abandoning subsistence agriculture (e.g., Díez, 2014; Kay, 2008; Köbrich & Dirven, 2007).

**FIGURE 1:**
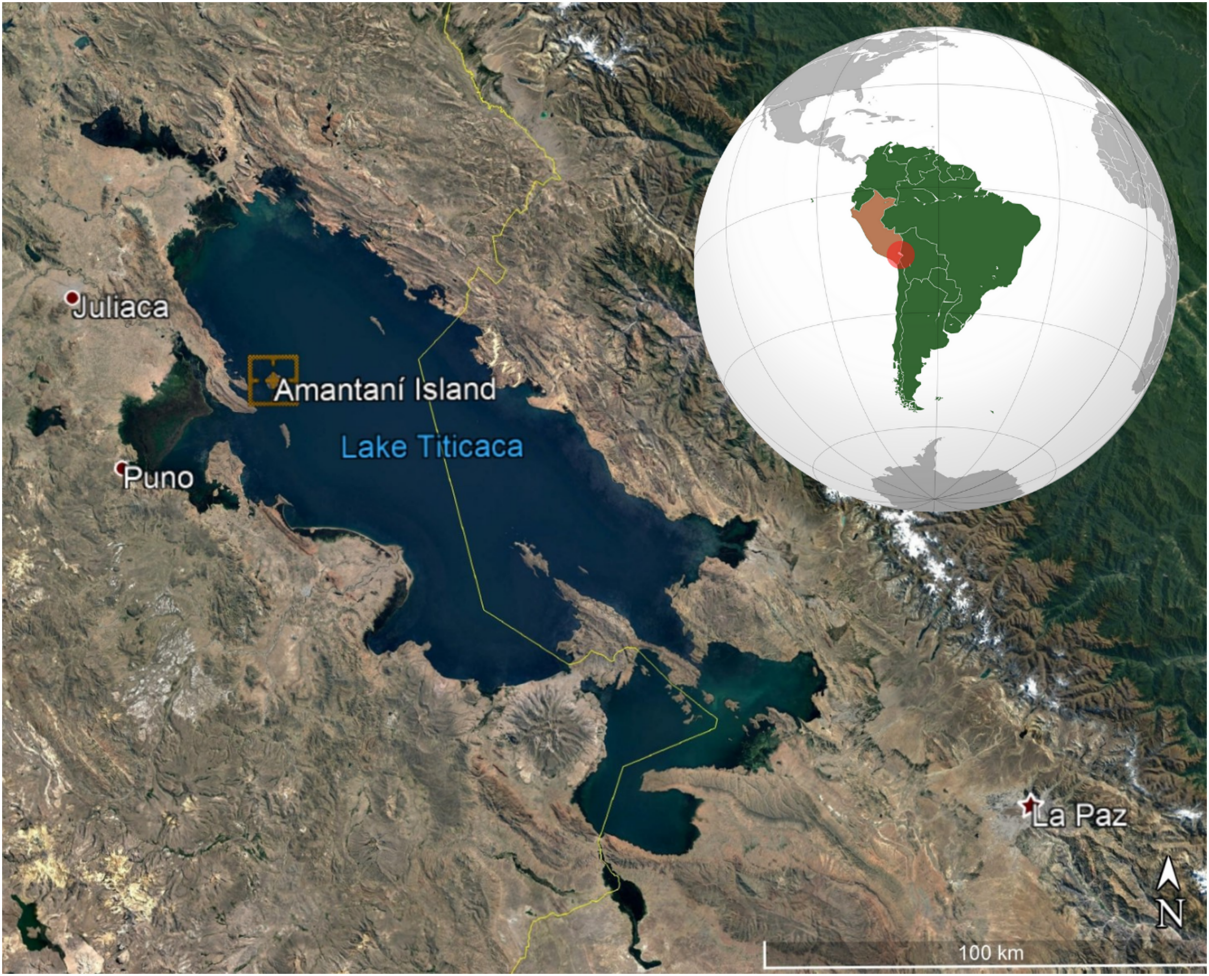
Location map of Amantaní Island. Source: Prepared by the authors

This article is framed within this line of research. We will analyse the case of Amantaní (Department of Puno, Peru), the most populated island on Lake Titicaca, whose inhabitants are Quechua peasants farmers (Figure 1). The emergence of new labour and consumer markets, stimulated by the improvement of transport and communication (mobile telephones), has generated a heterogeneous repertoire of reproductive strategies. In fact, almost every household seems to take a different approach. These strategies are the result of the connections that the islanders have chosen or been able to establish with these markets. The aim of this article is to examine the vulnerability and strength of these different pluriactive strategies and to discover the role that family farming resources play in this.

The COVID‐19 pandemic and the health measures established by the Peruvian Government between March and July 2020 offer the possibility of discovering hidden social aspects (Asensio, 2020). In this regard, we believe that these factors have acted as a stress test that allows us to glimpse the socio‐economic resilience of different pluriactive strategies; that is, not only their resistance to a sudden and severe market depression but also their ability to adapt to this new crisis context. Specifically, we are examining which kind of pluriactive strategies were most resilient when faced with the *tour de force* that was the pandemic crisis.

Let us answer this question right away: strategies in which subsistence agriculture played an important role. Households whose strategies depended on activities outside the island, which prevented them from exploiting their agricultural resources, were more vulnerable during the pandemic. The inclusion of subsistence agriculture in pluriactive practices is an aspiration of many islanders. Re‐peasantisation or re‐agrarianisation is sometimes seen as a response to a hostile market. But this has not been the case with Amantaní. On the contrary, agricultural activity increased as tourism revenues increased. The rise of community‐based tourism since the early 2010s encouraged many to adopt pluriactive strategies that boosted the recovery and expansion of the agricultural frontier. Surprisingly, the returns on this expansion were far below the effort invested, but it allowed the available domestic labour force to work and the family economy to be strengthened.

Our study is conducted from the paradigm of socio‐ecological resilience. Socio‐ecological resilience is the ability of a system to adapt to change. A socio‐ecological system is resilient if it maintains the connection between its elements in that transformation process. And that depends on its social capital and inclusion mechanisms (Adger, 2000) and on the flexibility and adaptive capacity of its community (Olsson et al., 2004) and domestic organisational structure (Díaz‐Aguilar & Escalera‐Reyes, 2020; Ruiz‐Ballesteros & Ramos‐Ballesteros, 2019). The use of this concept to study tourism in rural areas, and community‐based tourism in particular, has gained strength over the last decade (e.g., Cochrane, 2010; Espeso‐Molinero & Pastor‐Alfonso, 2020; Lew, 2014). Its usefulness is due to the fact that tourism involves transformation: changes in the production model, adjustments to organisational structures, and new uses of the ecosystem.

Lake Titicaca is part of the main tourist circuit of the central Andes, and this has led to community‐based tourism to become an important source of income in Amantaní. Community‐based tourism has been described in various ways, not always concurrent. We define it based on the concept of communal resources. The term “communal” refers to the management system and ownership of property. Communal resources are a source of income or benefits owned by an entire community without the exclusion of some or any of its members, which is reflected in more or less structured regulations. However, rural studies have shown that communal resources are far from materialising an idealised equitable and homogeneous society. On the contrary, they are functional to individual interests (Beltrán & Vaccaro, 2017). The unequal distribution of common benefits in the Andean region has been extensively studied (e.g., Golte, 1980; Smith, 1978; Urrutia et al., 2019). However, community‐based tourism often presents itself as an activity that aims to favour the whole community in a relatively egalitarian way. We understand community‐based tourism to be a small‐scale form of tourism, established in rural areas, in which the local population, through its organisational structures, plays a significant role in its control and management, to the point of restricting the participation of outsiders in this activity (Gascón & Cañada, 2005). But this does not mean that all members of the community have access to the benefits of tourism, or that these cannot be monopolised by one sector, as was the case in Amantaní for decades.

## 2 METHODOLOGY

An ethnographic methodology based on a deductive approach (Bernard, 2017) has been used in order to discover and identify the social behaviour of the Amantaní community. This method has allowed individual perspectives to be analysed in relation to their social, historical, and ideological context. The research techniques used have been qualitative in nature. They include participative observation, the carrying out of semistructured interviews (more than 300), life histories, informal conversations, and a retrospective evaluation of field diary entries. Following the interviews, participants were asked to provide their informed consent to the disclosure of their answers in social research. Their personal details would nevertheless be kept confidential. Quantitative surveys were also carried out, as well as a review of five decades of minutes of communal assemblies and the archives of different administrative institutions in Amantaní: the Governor's Office, the Municipality and *Sargento de Playa* (the institution in charge of controlling lake transport). Perusal of these documents helped understand the historical process of decision‐making in the tourism sector.

The analysis was longitudinal, with the authors completing several stays in the field over more than three decades. The first ethnographic work was carried out between 1990 and 1995 by the first author. Subsequently, various stays were made in later years. Finally, both authors returned to the territory in 2019. The second author carried out fieldwork during the COVID‐19 pandemic. Living in the city of Puno, he had daily access to islanders living in the city and travelled to the island on several occasions, when governmental health measures allowed it.

## 3 1980S: TEMPORARY EMIGRATION AND LOSS OF THE AGRARIAN FRONTIER IN AMANTANÍ

In the past, there were huge amounts of crops. There were potatoes, oca (Andean tuber)… The stores were full. There was enough to eat. In medium‐sized *chacras* (agricultural plot) there was a lot of oca. This year I visited one of those *chacras* and not a single arroba was harvested. Now they do not produce anything. (AM, 1995)

Throughout the rural area of the Andes, the diversification of economic activities among the peasant population has been a recurring phenomenon since the last third of the previous century (Bretón Solo De Zaldívar, 2015; Mesclier, 1993). In Amantaní, population growth—between the mid‐20th and late 20th century, the population here rose from around 1500 to 4000—the difficulty of expanding the agrarian frontier, and a culture based on the distribution of land through inheritance shared out between all a couple's children led to the creation of extremely small plots of land or microholdings. Currently, few peasants have more than half a hectare. By the 1960s–1970s, land did not meet the basic needs of most households, forcing agricultural yields to be supplemented by other economic activities.

In the 1970s, the community of Amantaní opted for tourism as one of those avenues for diversification. But travellers coming to the island were scarce throughout the 20th century. In addition, this limited tourism was monopolised by a certain group of peasants: the owners of the boats connecting the island to the mainland and transporting visitors (Gascón, 1996, 2005). Other nonprimary activities, such as the sale of basic products through grocery stores or the creation of handicrafts for the lean tourism that visited the island, were also not particularly significant. While those who ran a shop or received tourists had a more comfortable economic situation, they formed a small minority. The sale of handicrafts was managed cooperatively, but the low number of tourists in relation to the island's population meant that family income for this was occasional and insignificant.

In Latin America, and specifically in the Andes, pluriactivity is closely related to migration processes (Burneo & Trelles, 2019). Amantaní has been no exception. In many cases, migration was definitive. But in the 1980s and 1990s, many families would emigrate temporarily. Some members of the household, usually the father and/or one of the older children who had not yet become independent, would leave the island to work during the months between harvest and planting season in the nearby cities of Puno and Juliaca, or even further afield.

Permanent emigration reduced the pressure on microholdings. The establishment of sharecropping agreements (*waqi*) between relatives or friends was widespread: those who remained in Amantaní farmed the lands of those who had left. However, many migrants did not establish such agreements. Sometimes, this happened because they were living a long distance away and could not easily harvest their part of the crop. In others, because they hoped to return at some point: a long fallow period could ensure a good harvest during the first few years after their return.

In contrast, temporary emigration generated tension on the agricultural ecosystem for various reasons. One was the interest of households in maximising their agricultural resources. For an indigenous peasant population with a low level of formal education, emigration was a difficult decision, even more so during a few critical years for Peru, the so‐called “Lost Decade,” characterised by a depressed economy and a scarce and arduous labour market (Gonzales de Olarte, 1998). The more that could be obtained from the island's agricultural resources, the less it would depend on emigration. The result was, in many cases, overexploitation of the best land.

At the same time, temporary emigration also had an impact on agricultural infrastructure. Amantaní is a very steep island: in just 9.3 km^2^, it goes from an altitude 3810 m at Lake level to 4150 m. For this reason, its agriculture is based on a system of terraces, pre‐Hispanic in origin, which allow crops to be grown on steep slopes. These terraces require continuous maintenance, which is usually done between harvest and planting. But those were the months when most islanders had temporarily emigrated, so these jobs were often postponed. The result was that an important part of the terrace system ended up collapsing.

As mentioned above, boat owners reaped most of the benefits of tourism. They benefited from the transit of visitors in their role as transporters. At the same time, their control over the transport of tourists also allowed them to monopolise the income from accommodation and catering: they housed the travellers they transported in their houses. This made them the most economically powerful group in Amantaní and kept them from having to leave the island to look for work. Being able to stay in Amantaní all the time, they were able to take good care of their land. However, this did not apply to boat owners growing their crops on terraced lands as the maintenance of the terraces was labour‐intensive and could only be done through communal labour (*minka*), which was virtually unfeasible given the temporary emigration of most of the islanders.

In the early 1990s, about 25% of arable land was eroded or lost through poor practices and infrastructure neglect (CIRTACC, 1991). Added to this were the underutilised, abandoned, or fallow lands that seemed to have been left for good by owners who had emigrated permanently, or for a very long period. Amantaní's agrarian frontier shrank between the late 70s and the late 90s.

## 4 2000S: BOOM IN TOURISM AND CHANGE IN PLURIACTIVITY STRATEGIES

The rota system started in 2010. Until 2008 or 2009, tourism worked normally. Whoever could get close to a tourist, took him home. Why was the rota system introduced? Because tourism had increased. That is why they introduced the rota system (…). The rota system was introduced by the Municipality. The Municipality manages the income from tourism. The Municipality is in charge of charging for accommodation and food, and pays us after 15 days. (…) The Ministry of Tourism sends representatives to check the rooms, the beds. And with a document, it certifies that all is in order. Those who want to accommodate tourists must go to the municipality to register. Those who do not offer sufficient quality cannot put up tourists. They are told to prepare better for the next year. (AVC, 2019)

From the second half of the 1990s, the number of visitors to Lake Titicaca and its islands increased significantly. Between 1992 and 2015, arrivals to the Department of Puno increased from 19,368 to 198,817. This meant an average annual growth rate of more than 10%. This trend slowed in the last half of the 2010s but still continued to grow. In 2019, more than 230,000 visitors arrived in Puno (Observatorio Turístico del Perú, 2021). This strong growth in inbound tourism was due to several factors including the increase in international tourism at a global level, the interest of the State in promoting this sector as a strategy to secure foreign currency, the strengthening of a Peruvian middle class that began to travel around the interior of the country, and the end of the armed conflict led by Sendero Luminoso guerrilla fighters and Government armed forces, which had decimated Peruvian tourism in the 1980s (Rendón, 2015).

In the specific case of Amantaní, improvements to the transport system also favoured an increase in the number of visitors. First, faster boats reduced the time it took to get between the city of Puno and the island. And in 2009, a more direct route was opened through the neighbouring Capachica Peninsula, which halved the time taken to get to Puno, the administrative capital of the department, and almost a quartered the time taken to reach Juliaca, the economic heart of the region.

But more relevant was the reorganisation of the way in which benefits from tourism were distributed. First, timidly, through the increase in the number of boats, which almost doubled between 1985 and 1995. The boats were co‐owned, so by 1995, there were 91 families dedicated to this activity. This meant a certain increase in the percentage of islanders who benefited directly from tourist accommodation, which was only 65 families a decade earlier. However, that number accounted for less than 13% of the total households on the island (Gascón, 2005). More significant was the establishment of a rota system in the provision of tourist accommodation.

At the beginning of the 2010s, the Municipality of Amantaní established an agreement with most of the travel agencies in Puno to distribute their tourists to the homes of the islanders through a rota system.

This was possible because boat owners lost political hegemony. Since tourism started developing until the late 1990s, almost all governors were boat owners. The Governorship was, at that time, the most important post in Amantaní. Two factors enabled *lancheros* to control the Governorship. The first is that it was a costly post: it entailed high ceremonial expenses and no compensation. Therefore, only islanders with sufficient economic capacity could assume it. Moreover, at the time, this economic capacity was only available to those who monopolised the profits from tourism. The other element that allowed them to control the Governorship was the system used for electing governors. The outgoing governor chose three candidates to replace him. The shortlist was presented to the community assembly, which chose the new governor from among the three candidates. The outgoing governor's strategy was to prepare a shortlist that suited the interests of the boat owners' group: the candidates were always *lancheros* or their close associates.

Two reasons explain the interest of boat owners in controlling the Governorship. One was to use the island labour force (communal work) and the resources managed by the Governorship to their advantage. A review of the Governorate's minute books between 1975 and 1995 reveals that most of the projects proposed by this institution coincided with the interests of boat owners. Either it benefited them as transporters (construction, extension, and maintenance of docks), or as main beneficiaries of tourism (campaigns for the promotion of tourism, refurbishment of infrastructures and monuments aimed at promoting tourism, etc.).

The other reason for the boat owners' interest in controlling the governor's office was to counter opposition from certain sectors of the community to their monopoly over tourism. From this institution, they could hinder any attempt at regulation that sought to impose an equitable distribution of tourist accommodation. In the 1980s and 1990s, there were two attempts to establish a rota system in tourist accommodation. Both attempts failed due to the *lancheros*' control of tourist transport but also to the political backing of the Governorship.

However, by the early 2000s, the Governor's office was no longer the most important public institution in Amantaní. It had been superseded by the Municipality, where office is subject to elections. The rise of the Municipality at the expense of the Governorship was possible thanks to the increase of budgets through transfers from the central government. This was a process that occurred throughout Peru (Remy, 2005). As a result of this process, the Governorship, controlled by the *lancheros*, lost its importance. By the end of the decade, its role was reduced to transmitting the Municipality's orders and projects to the 10 communities into which the island is divided. Finally, the neo‐liberal policies implemented during the Fujimori government (1990–2000) put an end to the *lancheros*' monopoly over lake transport to the islands. This transport monopoly had been made possible by laws that favoured indigenous rights over the territory. However, boats from tourist agencies in Puno were now allowed to come and go to Amantaní carrying tourists. The local boat sector began to lose its prerogatives. This allowed the municipality to establish the tourist accommodation rota system.

The tourist accommodation rota system operates at two levels, first, among the 10 peasant communities into which Amantaní is divided. When it is the turn of a particular community, all tourists from the travel agencies are taken there. Then the second level of rotation comes into place, among islanders from that community who participate in the system. Once all the islanders in that community have received the established number of overnight guests, the system rotates to the next community.

By the early 2020s, the rota system had expanded the percentage of islanders benefiting from tourism. Approximately 55% of the islanders hosted visitors. Nevertheless, a substantial part was excluded, or excluded themselves, from the benefits of tourism. This sector was very heterogeneous. Some were elderly. Some did not want to accommodate visitors, but others were pressured into not participating in the rota system, although their homes met the habitability standards set by the Ministry of Tourism. It was felt that they were not offering a good service to tourists. The aim was to reduce the number of participants in the rota. This exclusion was legitimised in two ways, on the one hand, arguing that all islanders over the age of 65 already received a State pension “Pension 65”, aimed at elderly people living in poverty, and on the other hand, because various tour operators refused to take their tourists to certain homes and threatened to abandon the rota system if they were forced to. New couples who were still building their home—a process that can take years—were also excluded from the rota. The same was true for the islanders who had two homes and could not be in Amantaní when their community was rostered. Finally, some islanders who had other well‐paid sources of income and who, directly or indirectly, also benefited from tourism were also not involved. This was the case with store owners who serve visitors due to their location in the most central areas of the island, or of some of the master builders who work specifically to get houses ready to meet tourist requirements.

The distribution of benefits among tourist‐hosting islanders was also very uneven. Some had a more established infrastructure and bilateral agreements with travel agencies in Puno, Lima, Cusco, and even internationally. This minority was also better connected to the new marketing line of websites such as Booking.com, which directly connect the hosting service with the guest. Most of these successful entrepreneurs were former boat owners and their children who had raised start‐up capital when they monopolised tourism. In addition, they had experience in the tourism business and had established contacts and alliances with travel agencies. This sector of islanders, to a greater or lesser extent, have benefited most from tourism. On the other hand, the majority earn an income from tourism solely through participation in the rota system.

## 5 EXPANSION OF THE AGRARIAN FRONTIER

There are Amantaní people s who live in Lima for a long time. And then, one day, they suddenly come back to find that their brother is working the land that had been his before he left for Lima.. He complains, and goes to Puno to report his brother to the authorities. Then the justice system says: “you are no longer in a position to claim that land, because too many years have gone by. It no longer belongs to you. The land that was yours is now being farmed by your brother.”

So there are quite a few conflicts with migrants who want to get their land back?

That is right. Quite a lot. Moreover, I have to reconcile the parties, to mediate between them.

In your role as justice of the peace, what is your job?

Emigrants have rights. I have to determine whether the land belinged to the claimant before he left Amantaní and loaned it to his brother before he left for Lima or whether he had inherited after his departure. Therefore, I have to ask: “Did you farm on your land before you left for Lima?” And if he says “yes”, I have to ask his family and neighbours to see if it is true. If they say it is true, then he has a right to the land. If they say no, then it is up to them to solve the problem. Many men leave for Lima without ever cultivating their land. Then, they return and want it back. (SM, 2019)

The higher the participation in tourism activities, the lower the contribution made by agriculture to family income. But this is not accompanied by disinterest in agricultural activity. Paradoxically, the opposite is true. Two trends have been observed over the past two decades. One, at the family level, is the interest displayed by most households, especially those involved in the tourism sector, in recovering land that they had underutilised or lost, and even in increasing their lands. The other, at the community level, is the result of the sum of these individual interests: the expansion of the agricultural frontier.

This phenomenon is the result of four strategies. First, most of the agricultural terraces that had been lost have been recovered or are being recovered, and those that were still in operation are properly maintained.

Second, land that had never been devoted to agriculture, or that had long been lost, is being rotated. This is the case of Patapampa, the plateau that crowns the island, located at an altitude of more than 4000 m above sea level, and which occupies about 240 hectares. It was a space traditionally destined for grassland, but it was first cultivated in the late 2000s. This was made possible by climate change, which is very decisive in the Andean region (Pérez et al., 2010), which has reduced the impact of frost in that area. These are low yield lands: nonirrigated, stony, and battered by strong winds and small tornadoes that rip off its fertile layer. As a result, it must be left fallow (*wasara*) for periods of several years after one or two agricultural cycles. However, in mid‐2019, we estimated that four fifths of this area was being cultivated or had been and was now fallow. In the mid‐2010s, taking advantage of the fact that the water level of Lake Titicaca has decreased since the mid‐2000s (Chuchón & Pereira, 2017), the land surrounding the lake began to be recovered. In September 2019, we estimated that some 2.5 hectares of a narrow stretch of shoreline ranging from 0 to 15 m wide had been gained. Although these lands are good quality and easily irrigated, their recovery was very laborious, because they were covered with stones, sometimes up to a metre deep. Rotation involves effort greater than the benefits it can offer in the medium term. In addition, there is a risk that, at any time, the lake will return to the levels of the past.

The third phenomenon that reveals interest in improving agricultural infrastructure is the active guano market established with Ccotos, a nearby shoreline community dedicated to sheep farming. Sacks of guano piled up on the docks have become a constant image in the months prior to the planting period. Initially, it was the Amantaneans who had to hire a boat to go in search of this agrarian input. The market has become so powerful that Ccotos farmers themselves, or intermediaries from the Capachica Peninsula, who previously buy the wet guano and dry it out, now transport it to the island. In the past, some islanders would occasionally buy industrial fertilisers, but they were expensive. The traditional way of recovering the productive capacity of the land was based on leaving the land to rest for a few years or it depended on the manure provided by the scarce island cattle.

Finally, the fourth phenomenon is the emergence of a timid land market. On the supply side, the market draws on the interest of permanent emigrants in selling off their lands. On the demand side, some households are interested in reversing the effects of inheritance division. Normally, these processes are channelled within the extended family: between siblings and cousins. When this is not the case, only those with greater purchasing power—such as those with stronger connections to the tourist market—can buy land. These islanders are always eager to acquire any plot of land that is for sale.

But what is the interest in investing labour and capital in agricultural activity when it seems unprofitable on the basis of income and expenditure calculations, especially when its role per household is becoming less and less decisive in a pluriactive economy. Several factors explain this behaviour. One is that income from tourism is not sufficient to ensure reproduction for most families. Subsistence agriculture continues to play an important role in the domestic economy: it supplies the household with basic foods for much of the year (López de Romaña, 2019).

Another factor is due to the periodic nature of tourist activity and the fact that its management based on the accommodation rota system. Participating in tourism involves two things in relation to the family workforce. On the one hand, tourist accommodation is not a daily or constant activity. There may be weeks or months between rotations, depending on whether it is high or low season. On the other hand, it is difficult to combine tourism with occupations outside Amantaní: you have to be on the island to accommodate visitors when you are rostered, and you never know exactly which dates it will be. There are some islanders who can negotiate jobs away from Amantaní with this unstable seasonality, but most cannot. Therefore, entering the tourism rota system implies, for most households, choosing to reside on the Island. So, what to do with the available family workforce, when tourism only uses it intermittently?

The expansion of the agricultural frontier, even with low yields and a strong labour investment, is a way to maximise domestic labour. In a peasant economy, calculations are not based on the relationship between investment and profit. The objective is to provide work for the entire available workforce, even if yields are lower than production costs (Chayanov, 1974). The availability of domestic labour is not elastic: regardless of whether they are working or not, the family must ensure the reproduction of all its members. It is therefore more useful to devote this workforce to activities with low yields than to leave it idle.

Finally, devoting efforts to agriculture is a strategy of resilience: the people of Amantaní cannot control the labour market outside of the island, or tourist flows, or the price of domestic consumer products, but they can control agricultural production for their own consumption. It should be noted that this strategy is found in families across the socio‐economic spectrum in Amantaní. The COVID‐19 pandemic has highlighted this situation.

## 6 THE COVID‐19 PANDEMIC: A STRESS TEST FOR THE RESILIENCE OF ECONOMIC STRATEGIES

The government has decreed a curfew and total confinement from 8 p.m. onwards. However, migrants living in Puno and Juliaca return to Amantaní after that time. They return at night, so as not to be detected. They arrive in Capachica, I do not know how. From there, by boat, to Amantaní. (…) There were people who had left many years before and who nobody knew on the island. As soon as they arrived, they looked for their land and started to work on it. (HC, 2020)

The COVID‐19 pandemic has been a test of the resilience of the islanders' various economic strategies. The pandemic has provided a glimpse of their resilience not only with respect to a sudden and severe market depression but also in terms of adapting to the COVID‐19 crisis.

Peru was one of the South American countries most affected by the pandemic. Initially, the impact was relatively small in the Andean region. Even in February 2021, no cases of infection had been detected in Amantaní, nor was there any suspicion that anyone could have had the disease. However, in mid‐March 2020, the government established a series of nationwide mandatory emergency measures, drawing no distinction between natural regions, or between urban and rural areas. These measures included the closure of borders and lockdown, which was called “compulsory social isolation.” As of July, these measures were relaxed in less affected departments, such as Puno. But this did not activate tourism: the international tourist market was stagnant, and domestic tourism remained scarce. Furthermore, lockdown, which led to a ban on movement from one area to another, made dual‐residence strategies difficult.

Lockdown also adversely affected production and commercial activity considered nonessential. Many islanders who lived away from the island or has dual residence were left without work. One of the most affected activities, and in which many emigrants from Amantaní participate, is alpaca hide and leather handicrafts. This is a job that depends on tourism, which is its main market. The state of emergency led to the closure of these workshops. We will return to the subject of leather artisans later.

The paralysation of tourist activity was an economic debacle for the islanders, but not to the same degree: it depended on their strategies of pluriactivity. Households that included agriculture were more resilient. Subsistence agriculture provided these families with a substantial part of the basic foodstuffs required in their diet. In addition, the emergency period began shortly before the harvest, which takes place between April and June. This assured them of food, at least for a few months.

They also activated barter strategies, which had previously disappeared. Traditionally, the islanders offered wood and artisanal stone utensils to these markets, and in return, they obtained basic food products (potato, oca, chuño, etc.). An economic logic based on pluriactivity and monetarised revenues marginalised these trades in kind. Amantaní is dotted with small quarries of a tough stone that is relatively easy to work with, from which many families made different utensils (metates, chairs, etc.) that were in demand in those barter markets. By the end of 2019, most of these quarries were abandoned, and few islanders made stone crafts. Practically all of the inhabitants of Amantaní grow eucalyptus to meet their construction needs, but which, in the past, went to the barter market. The pandemic's disruption of monetarised income‐generating activities prompted the recovery of trade in kind with different farming communities along the shoreline of Lake Titicaca.

Households that had emigrated temporarily or definitively or who had dual residence without engaging in agriculture faced greater problems. Faced with the paralysation of their economic activities, many decided to return to Amantaní. It was a strategy that became widespread across the country (Burneo & Castro, 2020; Burneo & Trelles, 2020). In the community of Pueblo alone, the most populated of the 10 communities that make up the island, 37 families returned in the first weeks of lockdown. They immediately began to recover their land, which in some cases had been leased and in others neglected. Many of those who returned were young people who had not even been born on the island and who had to ask where their agricultural plots were located. But agriculture is a parsimonious activity: they had to prepare the land, plant, and finally, wait for their crops to bear fruit, a process that could take more than a year, taking into account the point in the agricultural cycle when they returned. They also did not have any material to barter: they did not know the art of stone carving, and if they did have any eucalyptus trees, these had not been taken care of. Eucalyptus trees need to be pruned regularly. Otherwise, the wood grows with knots that reduce its strength and render it useless as a construction material. The initial ban on any movement also made it difficult for these people to access the monetarised food market: weekly markets on the island, organised by traders from Capachica, were interrupted, and trips to external markets were made in semiclandestine conditions (López de Romaña, 2020).

Why did that population decide to spend lockdown in Amantaní when many had left years ago? And why did they begin to recover agricultural activity, when emergency measures were not expected to last as long as the 2022 harvest period?

Several factors explain this strategy. On the one hand, the costs of reproduction: living in the city is expensive. Most emigrants live in rented houses, whereas in Amantaní, they had the family‐owned home. In addition, in the city, basic goods and services have to be purchased, and now they had run out of income. On the island, on the other hand, systems of help or exchange between relatives, friends, and neighbours can be activated, where money is replaced by a long‐term loan or labour.

The recovery of agricultural activity can be explained by two reasons. On the one hand, in Amantaní, it became the only working activity available for the domestic work force. Lockdown was expected to end well before the 2022 harvest, and it was thought that the population would return to its economic activities in the city. But that agricultural work could be profitable: lands that had been prepared and perhaps even planted could be leased to a neighbour or family member or a sharecropping agreement could be reached.

The other reason for the return of emigrants to the island and their recovery of agricultural activities is the desire to change their pluriactivity strategy. This was an aspiration that existed before, and which the pandemic accelerated. Let us return now to the case of the leather artisans.

## 7 LEATHER ARTISANS

Older leather artisans were the first who learned the trade. They taught others and took young people to Lima. Gradually, many became leather artisans. In Lima, most leather artisans are from Amantaní (AC, 1994).

In Lima there are 3,000 Amantaní people. They have a reputation as the best leather artisans. (JM, 1992)

Emigration is a highly structured strategy, which is carried out through networks of relatives, friends, and neighbours (Adams & Valdivia, 1991; Paerregaard, 2017). The case of Amantaní is paradigmatic: this system of networks has allowed many islanders who emigrated in the 1980s to dedicate themselves to the artisan manufacture of alpaca hide and leather goods for the tourist market and export. This is surprising given that alpacas have never been bred in Amantaní. It was a pyramidal process. First, a small core group of emigrants from Amantaní learned the trade, attained a degree of mastery, and set up their own workshop. As the demand for these crafts grew, they hired others from Amantaní as apprentices. As these, in turn, became independent, they also sought others from their homeland to provide labour (Gascón, 2004).

By the beginning of the 1990s, many of these leather artisans had returned to the island. They had established trade partnerships, and they did not need to live in the city to do their job. Transporting the raw material to the island was difficult and costly, but it was compensated because life in Amantaní was cheaper and they could combine their artisan work with agriculture. From time to time, they would travel to Juliaca or Lima to deliver the finished products to their customers. With the boom in tourism, demand increased, and that encouraged a certain degree of technification in the craft process. But the new tools required a resource that did not exist on the island: electricity. In order to stay connected to the leather market, production times had to be cut, and therefore power tools had to be purchased and used. Some leather artisans chose to leave the trade. They invested their capital in improving their homes and providing tourist accommodation. But others returned to the cities, especially Juliaca and Lima, to continue working as leather artisans. By the mid‐2010s, only a few leather artisans still lived on the island.

Getting electrical infrastructure in place had been a goal for the people of Amantaní since the 1970s. Over the next half century, various projects failed. It was finally achieved at the end of 2019, a few months before the state of emergency was declared. The construction of the infrastructure, based on a complex and cumbersome system of solar panels, took several years. The arrival of electricity encouraged many leather artisans who had previously emigrated to consider returning to Amantaní, and during those years, they began to renovate their old family houses. Improvements in the transport system, which made it easier and cheaper to transport raw materials and finished handicrafts between Amantaní and the market, also contributed to this, as did the arrival of mobile telephones, which allowed for immediate contact with suppliers and customers.

The fact that a substantial group of leather artisans who had emigrated wanted to return to Amantaní is accredited by two factors. On the one hand, the deputy‐governors of each community carried out a double census: one registering the homes of families considered full members of the community through their participation in community activities and another registering houses that had requested connection to the new electrical system. The two censuses did not coincide: the second was more numerous. The second factor was the composition of the population that returned when the state of emergency was declared. In the case of Pueblo, of the 37 families that returned, 24 were involved in leather handicrafts. In the neighbouring community of Lampayuni, the percentage was similar: 70%.

Some of these leather artisans, especially those who had no direct family on the island, returned as a temporary safety measure, and left in July, as soon as lockdown was lifted. But others were leather artisans who had been preparing for their return for a long time when electricity finally became a reality. Their objective was to change their pluriactive strategy: to combine leather handicrafts with tourism, joining the accommodation rota system, and to gradually recover their land and agricultural production. The pandemic caught them by surprise before they could make the change, with the first two activities interrupted, and without having initiated the third. They returned earlier than planned, and without anything else to do, they went straight to working the land.

## 8 CONCLUSIONS

The case of Amantaní reveals three characteristics of peasant pluriactivity: plurality, adaptability, and universality. By “plurality,” we mean that pluriactivity is not a univocal option either. In Amantaní, as new employment and economic possibilities have emerged, the disparity of pluriactive strategies has become accentuated. Some households decided to stay on the island most of the time, taking advantage of the boom in tourism and increasing the profitability of agricultural activity. Others, on the other hand, opted to rely more on the external labour market, to practice long‐term emigration, and to abandon subsistence agriculture. And between these two extremes, we have seen a whole host of different strategies.

By “adaptability,” we understand that this is not a static strategy. Households are always willing to change the intensity and nature of their relationship with the modern market (Van der Ploeg, 2008, 2013). In the case of Amantaní, this has depended on factors such as the short‐term conditions of the labour market, their level of training and education (which makes it possible to take advantage of this market in one way or another), the development of communication technologies (ICT) and means of transport, the available energy sources, the development of tourism, the investment capital they have accumulated, and the strengthening or weakening of the national economy. Returning to Chayanov, and although we have not discussed it in this article, the mutability of these strategies also depends on the phase of the family cycle. The average age of its members, their educational needs, and the availability and characteristics of the domestic workforce are factors that determine whether one pluriactive strategy is chosen over another.

In Amantaní, diversification of economic activities is pursued by both impoverished households, which have to address the scarcity of land by accessing other sources of income, and by people with some capacity for capitalisation who see these farming activities as an investment option (Dandler, 1987). This is what we mean by “universality”: regardless of the socio‐economic situation of the household, they all engage in pluriactivity. The decision regarding pluriactivity is made based on several aspects. These include the opportunities that the modern market, or the networks of economic interests that the household has been able to establish, may offer in the short term. But the possibility of providing work to the available domestic work force and the desire to ensure resilience are also important. These latter two factors explain why, in Amantaní, efforts to recover the agrarian frontier are not only concentrated in households that cannot meet their reproductive needs through income from tourism Families that are most successful in tourism are equally interested in subsistence farming. In their case, the percentage of family income represented by agricultural activity is lower than for the rest of the islanders. Nevertheless, they are making efforts to enlarge their holdings, even if yields are lower than the effort invested. They also acquire other land if they have the opportunity.

We must ask ourselves whether community‐based tourism is an activity that facilitates resilience. There is much debate surrounding this subject (Milano & Gascón, 2017). Some authors argue that, if effectively managed, it is an activity that can improve peasant incomes, strengthen the community, and improve market connections (Moscardo, 2008; Ruiz‐Ballesteros, 2011). Others, on the other hand, note that it can often create a mirage of development, increase internal conflicts and socio‐economic differences, and/or establish dependency on the intermediary agents who control the influx of tourism (Blackstock, 2005; Gascón, 2013).

Beyond the income it can offer, tourism is a vulnerable activity: it depends on an external market that cannot be controlled, and which can be interrupted by factors such as political instability, trends, or a pandemic (Cañada & Murray, 2021; Mowforth et al., 2008). But in the case of Amantaní, the type of tourism and the management system adopted from the mid‐2010s onwards have favoured the spread of pluriactivity strategies that reduce this vulnerability. These are strategies based on subsistence agriculture. The tourism accommodation rota system increased the number of islanders who were able to participate in tourism, but at the same time required them to live on the island, or to reside there for much longer periods of time, reducing or bring to an to end their stays in the city. Given the lack of other activities for the domestic work force, they devoted their time to recovering, improving, and expanding their agricultural land. Subsistence agriculture for the purpose of self‐consumption not only allows the domestic work force available to be employed, even with negative yields, but also provides a safety net. This was observed from March 16, 2020, when the Peruvian government declared a state of emergency on account of the COVID‐19 pandemic. Households that combined tourism, or other economic activities, with subsistence agriculture for self‐consumption were more resilient: they adapted better to the economic crisis brought about by lockdown and the closure of borders.

Therefore, greater connectedness to the monetarised and nonagricultural labour market does not necessarily lead to depeasantisation. It depends on the possibilities offered by that market, the options available to peasants to access it, and the strategic decisions of each household. The combination of these and other factors results in a range of pluriactivity options, in which the role of agriculture for self‐consumption may differ. And as far as possible, the islanders take an approach to pluriactivity that includes this agriculture. We have seen this in the case of the leather artisans who were already preparing for their return before the pandemic hit, taking advantage of the new electrical infrastructure that would allow them to relocate their workshops to the island. It should be noted that the case of Amantaní is not exceptional. Other case studies have already shown that, often, the greater the pluriactivity, the greater the interest there is in devoting labour and capital to agricultural activities as a strategy of resilience when faced with uncertainty (e.g., Aguilar‐Støen et al., 2016; Narotzky & Smith, 2006; Winkel et al., 2016).

The case analysed here brings two elements to the analysis of agrarian change, when tourism is involved in this change. On the one hand, rural tourism cannot be studied in isolation when it is part of a pluriactivity economy—even if the analytical interest is tourism. In a pluriactive economy, no source of income is independent or autonomous. They are part of an overall strategy of optimising resources—land, capital, social networks, expertise in each economic sector, and so forth—aimed at meeting family needs and occupying the available workforce. On the other hand, tourism cannot be evaluated solely on the basis of the short‐term income it brings to the rural household economy. This is a sector prone to crises outside the control of the local population. Amantani's tourism history has shown this vulnerability: political violence of the 1980s, COVID‐19 pandemic in the 2020s. It is therefore necessary to consider its role in the long term. The role of tourism in the resilience of the rural economy must be valued.

In fact, the pandemic further convinced islanders of the need to seek resilience in pluriactive strategies that included agriculture. For decades, and with the approval of the majority of islanders, the main obsession of the Municipality had been to devote community efforts and public budgets to promoting tourism. With the same consent, at the end of June 2020, this institution decided to implement productive projects, such as bio‐allotments and fish farms, and to postpone actions aimed at improving tourism infrastructures. Amantaní was similarly not an exception in this regard. Emergency studies published during the first year of the pandemic showed that agriculture, compared with other economic activities, and in particular peasant agriculture compared with agro‐industry, was more resistant to crisis and lockdown (Altieri & Nicholls, 2020; Van der Ploeg, 2020; Worstell, 2020).

The first lockdown—another was decreed in 2021 on account of the worsening of the pandemic—was not easy for those who had returned and whose pluriactive economy excluded subsistence agriculture for self‐consumption, and not just because they lacked cash and income in kind and also because they were rejected by permanent residents. In the short term, these permanent residents saw the food aid received by the State dwindling as it had to be distributed among the newcomers as well. In the medium term, they were seen as a factor that would accentuate the phenomenon of microholdings and halt efforts to increase land per household. If they had abandoned their plots of land for years, these lands would no longer enter the land market, or they would have to be returned if a neighbour or family member had exploited them surreptitiously. If they had established sharecropping agreements, sharecroppers would lose the usufruct of these lands.

Peru was one of the South American countries most affected by COVID‐19. Several pandemic waves hampered the revival of its tourism sector. In mid‐2021, few visitors were coming to Amantaní. Some of the islanders chose to return to the cities, to seek work in essential sectors that were still functioning, or to mining or intensive agriculture areas. However, those who stayed pursued a strategy of self‐sufficiency based on farming and on the use of family labour to clear new land.

## Data Availability

No quantitative datasets were generated or analysed. Qualitative data supporting the findings of this study may be shared upon reasonable request from the corresponding author. However, due to ethical restrictions, privacy and anonymity are prioritized.
